# Novel Stemness-Associated Scores: Enhancing Predictions of Hepatocellular Carcinoma Prognosis and Tumor Immune Microenvironment

**DOI:** 10.32604/or.2025.063993

**Published:** 2025-07-18

**Authors:** Gaofeng Pan, Jiali Li, Weijie Sun, Jiayu He, Maoying Fu, Yufeng Gao

**Affiliations:** 1 Department of Infectious Diseases, The First Affiliated Hospital of Anhui Medical University, Hefei, 230022, China; 2 Anhui Province Key Laboratory of Infectious Diseases, Anhui Medical University, Hefei, 230022, China; 3 Department of Infectious Diseases, The First People’s Hospital of Kunshan, Kunshan, 215300, China; 4 Department of Medical Oncology, First Affiliated Hospital of Bengbu Medical University, Bengbu, 233099, China

**Keywords:** Hepatocellular carcinoma (HCC), stemness, prognostic model, machine learning, TOMM40L, vitro experiment

## Abstract

**Aims:**

The aim of this study is to develop a prognostic model for hepatocellular carcinoma (HCC) using stemness-related genes (SRGs), while also pinpointing and validating pivotal genes associated with this process.

**Methods:**

Utilizing the TCGA and ICGC database, a prognostic stemness-related scores (SRS) for HCC through a combination of WGCNA and machine learning. Bioinformatics analysis evaluated tumor immune infiltration characteristics and drug sensitivity in different SRS subgroups, identifying the key gene TOMM40L. qRT-PCR and IHC were employed to detect the expression level of TOMM40 L. Kaplan-Meier survival analysis assessed the prognostic value of TOMM40L in HCC. *In vitro* cell experiments explored the influence of TOMM40L on HCC cell progression and stemness.

**Results:**

The prognostic model SRS for HCC was developed and validated, incorporating four SRGs: EIF2B4, CDCA8, TCOF1, and TOMM40L. Distinct variations in tumor immune infiltration profiles and drug sensitivity were noted across different SRS subgroups. Elevated TOMM40L levels are notably detected in malignant tissues in contrast to adjacent tissues, with heightened TOMM40L expression correlating with unfavorable prognostic outcomes. In addition, knockdown of TOMM40L significantly inhibited cell progression and stemness. **Conclusion:** The newly constructed SRS model is a potential biomarker for assessing HCC prognosis, and the key gene TOMM40L exhibits oncogenic properties.

## 1 Introduction

Hepatocellular carcinoma (HCC) is a recognized global health challenge, particularly in Asia and sub-Saharan Africa. Although global HCC incidence appears to have stabilized [1], the five-year survival rate remains around 10% [2]. Postoperative five-year recurrence rates exceed 70% [3]. Traditional clinical and pathological parameters such as vascular invasion offer some prognostic guidance, but their efficacy in individualized prognosis, especially for chronic hepatitis B-related HCC, is limited due to the diverse and complex biological behavior of HCC.

Predicting HCC prognosis remains a research focus, yet current models and biomarkers are still unsatisfactory. Cancer stem cells (CSCs) are instrumental in HCC by contributing significantly to its initiation, recurrence, metastasis, and resistance to chemotherapy and radiotherapy [4,5]. Studies have shown [6,7] that CSCs in HCC exhibit significant heterogeneity, with specific genes in different subgroups independently associated with prognosis, indicating the important influence of CSCs on tumor progression and intratumorally heterogeneity.

This study seeks to systematically investigate the holistic significance of stemness-related genes (SRGs) in HCC and develop a robust and precise prognostic indicator, the Stemness-related scores (SRS), to forecast the survival outcomes of HCC patients. Further, we associated the SRS with the tumor microenvironment and drug sensitivity, identifying and validating the oncogenic role of key gene TOMM40L, providing new perspectives for HCC diagnosis, treatment, and prognosis.

## 2 Materials and Methods

### 2.1 Data Download and Core SRG Screening

Transcriptomic data (FPKM) for 50 normal liver tissues and 374 HCC tissues, along with clinical prognostic information such as age, sex, stage, pathological features, and Overall survival (OS), were obtained from the TCGA database. In this study, we conducted a systematic analysis of 4419 SCRG genes. Differential expression analysis was performed using the Limma package, with significance defined as |log_2_(foldchange)| > 1 and *p*-value < 0.05. Additionally, WGCNA is particularly effective in discerning complex gene-gene interactions and elucidating co-expression patterns, achieving a scale-free R^2^ = 0.9, which indicates a strong fit of the network to scale-free topology models [8,9]. This provides a robust framework for linking gene modules to clinical characteristics. In our research, we integrated WGCNA with differential expression analysis to identify key gene modules associated with the HCC phenotype, resulting in the identification of 1004 important stemness-related genes associated with HCC.

Subsequently, we further filtered these 1004 genes using the random forest algorithm. This method is commonly employed for feature gene selection in biological research [8,9]. The decision to filter key genes based on an importance score greater than 1 ensures the selection of genes that make significant contributions to the gene expression profile of HCC. This methodological integration not only allowed us to focus on the most relevant genetic factors but also enhanced the reliability and interpretability of our findings. Ultimately, 34 key genes were selected, of which 29 core genes were determined to be potential prognostic risk factors for HCC through univariate Cox (unv-Cox) regression analysis.

### 2.2 Development and Validation of the Prognostic Model

According to our previous study [8,9], we constructed model based on SCRG using unv-Cox, LASSO, and multivariate Cox (mut-Cox) analyses. The SRS = (EIF2B4 expression ∗ Coef) + (CDCA8 expression ∗ Coef) + (TCOF1 expression ∗ Coef) + (TOMM40L expression ∗ Coef). HCC cases were divided into high-SRS groups (HSG) and low-SRS groups (LSG) based on the median risk score. Prognostic value was assessed using “survminer” and “timeROC”. A nomogram was created using “rms” and “regplot”, and its accuracy and stability were evaluated by calibration curves, DCA, and ROC curves.

### 2.3 GO, KEGG and GSEA

Differentially expressed genes (DEGs) between HSG and LSG were analyzed using the “Limma” package (|log_2_FC| > 1, FDR < 0.05). GO analysis, KEGG analysis and GSEA was performed using “clusterProfiler”, “org.Hs.eg.db”, and “enrichplot” to explore biological differences between SRS subgroups.

### 2.4 Immunological Characteristics Analysis

The ssGSEA algorithm in the R packages “GSVA” and “GSEABase” estimated the infiltration levels of 22 immune-related cell types in HCC samples. Common Human leucocyte antigen (HLA) family genes and immune checkpoint molecules (ICM) were summarized and their correlation with risk scores was analyzed and presented in radar plots. In addition, we also explored the somatic mutation spectrum using the maftools package [10], listing the top ten frequently mutated genes in different risk score subgroups to investigate the immune landscape and genetic characteristics associated with the SRS subgroup in HCC.

### 2.5 Common Drug Sensitivity Analysis

The sensitivity of common drugs was assessed using the R package oncoPredict [11].

### 2.6 Sample Collection

This study included 40 patients diagnosed with HCC at the First Affiliated Hospital of Anhui Medical University (FAHAMU) from February 2022 to April 2023. All patients were free of other malignancies. RNA from fresh samples was stored at −80°C. Approval was obtained from the Ethics Committee of the FAHAMU (LLSC-2022424). Informed consent was obtained from all patients involved in the study. Formalin-fixed paraffin-embedded tissue microarrays (TMAs) containing 90 HCC and 90 adjacent tissues were provided by Shanghai Outdo Biotechnology Co., Ltd. (Shanghai, China).

### 2.7 Real-Time Quantitative PCR (RT-qPCR)

The RT-qPCR method was briefly described above [12]. TOMM40L mRNA levels in 40 fresh HCC tissues were detected. Samples were collected within 30 min of excision and quickly frozen in liquid nitrogen to prevent RNA degradation. Total RNA was extracted using Trizol reagent (Invitrogen, 15596026, Waltham, MA, USA) and reverse transcribed into cDNA with TAKARA reverse transcription kit (Takara Bio, RR036A, Kyoto, Japan). Fluorescent quantification was performed with TB-Green qPCR (Takara Bio, RR420A), using β-actin as the internal control. Human primer sequences were as follows: β-actin forward, CACCATTGGCAATGAGCGGTTC; reverse, AGGTCTTTGCGGATGTCCACGT; TOMM40L forward, TGGCGAGTATCGGGGAGATG; reverse, CTCCCTTAGTCCACAGCCAC.

### 2.8 Immunohistochemistry

The IHC method was briefly described above [13]. TMA with a thickness of about 4 µm was used to evaluate TOMM40L protein levels in cancerous and adjacent tissues. TMA underwent deparaffinization, rehydration, and antigen retrieval in 0.01 M citrate buffer (pH 6.0) with 100°C. Endogenous peroxidase activity was inhibited with 3% H_2_O_2_. Immunohistochemical staining employed a rabbit and mouse universal TOMM40L antibody (1:100; Bioss, bs-17317R, Peking, China), incubated overnight at 4°C. Negative controls used PBS instead of the primary antibody. After incubation at room temperature for 20 min, wash with PBS, then add the secondary antibody solution (1:100; Bioss, bs-0312R) dropwise on the tissue surface (complete coverage) and incubate at room temperature for 2 h. Use DAB colorimetric solution (ThermoFisher Scientific, 34002, Waltham, MA, USA) to develop color, and then use deionized water to terminate staining. Stained sections were scanned and analyzed using the digital slice viewer system (KFBIO, KF-PRO-020-HI, Ningbo, China). Immunoreactivity score (IRS) combined staining intensity and percentage of positive cells [14]. Two experienced pathologists independently determined IRS, categorizing TOMM40L expression levels as negative (-), weak positive (+), moderate positive (++), and strong positive (+++). ROC curve analysis determined high and low TOMM40L expression in cancerous and adjacent tissues [15]. Scores ≤ 4 indicated low expression, while >4 indicated high expression.

### 2.9 Cell Culture and Transient Transfection

HepG2 and HCCLM3 cell have been cultured long-term in our laboratory [8,9]. All cells were free of mycoplasma contamination after STR identification. Cells were cultured in DMEM medium (Invitrogen, 11965092) with 10% fetal bovine serum (Invitrogen, A5670701) and 1% penicillin-streptomycin (Invitrogen, 15140122). Transient transfection was following the manufacturer’s instructions (Polyplus, Shanghai, China). siTOMM40L sequences are listed in Table 1.

**Table 1:** siTOMM40L sequences

Name	Sequence (5^′^–3^′^)	
siNC	Sense	UUCUCCGAACGUGUCACGU
	Antisense	ACGUGACACGUUCGGAGAA
siTOMM40L-1	Sense	CCGUCUAUGCAAAGAUGUA
	Antisense	UACAUCUUUGCAUAGACGG
siTOMM40L-2	Sense	GGAUAGUAACUGGUGUGUA
	Antisense	UACACACCAGUUUACUAUCC

### 2.10 CCK8 Assay and Transwell Assay

In brief, CCK8 Assay: 1 × 10³ HCC cells were inoculated in a 96-well plate for culture, CCK8 solution (Bioogenetech, RK001099, Shanghai, China) was added at the specified time point, incubated for 2 h, and then the absorbance (OD value) was measured at a wavelength of 450 nm using a microplate reader (Biotek, ELX800, Winooski, VT, USA) and data analysis was performed. Transwell Assay: No matrigel was added to the upper chamber to explore cell migration ability, and matrigel (Corning, 356234, NY, USA) was added to explore cell invasion ability. 50,000 HCC cells were resuspended in serum-free DMEM medium and inoculated in the upper chamber of the Transwell chamber, and 800 μL complete DMEM medium was added to the lower chamber. The experiment was terminated after 24 h of culture, and the upper layer of cells in the chamber was wiped off with a cotton swab, fixed with 4% paraformaldehyde, stained with 0.1% crystal violet, and finally counted and statistically analyzed.

### 2.11 Colony Formation Assay

HCCLM3 and HepG2 cells were plated at a density of 1 × 10^3^ cells/well in 6-well plates and cultured for 12 days. Cells were fixed with formaldehyde for 25 min and stained with crystal violet for 20 min. Colony numbers were counted to evaluate cell proliferation.

### 2.12 Spheroid Assay

Five hundred experimental cells were seeded in ultra-low adhesion 24-well plates and incubated in DMEM/F-12 medium (Invitrogen, 11320033) containing 2% B27 (ThermoFisher Scientific, 17504044), 20 ng/mL recombinant human epidermal growth factor (EGF; ThermoFisher Scientific, AF-100-15-500UG), 20 ng/mL recombinant human fibroblast growth factor (FGF; ThermoFisher Scientific, 100-18B-50UG) and 1% N2 supplement (ThermoFisher Scientific, 17502048) for five days. The number of spheres with a diameter of more than 50 μm was counted under a microscope (Leica, DM6B, Wetzlar, Germany).

### 2.13 Statistical Analysis

mRNA and protein levels were expressed as Median (min, max). Differences in mRNA and protein levels between cancerous and adjacent tissues in HCC cases were compared using the rank-sum test. Differences in protein levels between groups based on clinical parameters were evaluated using the Mann-Whitney U test and Kruskal-Wallis’s test. Spearman rank correlation analysis was used to assess the correlation between protein levels and clinical indicators. Survival curves were plotted using the Kaplan-Meier method and survival analysis was performed using log-Rank. The Cox proportional hazards model was used to analyze hazard ratios (HR) for various prognostic factors. For *in vitro* cell experiments, paired *t*-tests were used to compare two groups. Statistical significance was set at *p* < 0.05.

## 3 Results

### 3.1 Screening for DEGs Related to Stem Cell Stemness

**Figure 1:**
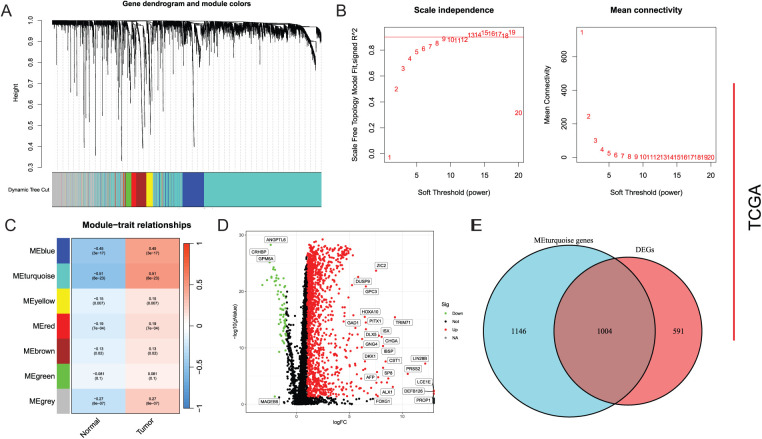
Screening of DEGs. (**A**) Mean connectivity and scale independence; (**B**) Dendrogram of gene clustering displaying seven identified modules; (**C**) Heatmap of module-trait relationships; (**D**) Differential analysis depicted in a volcano plot; (**E**) Common genes between key module genes and DEGs showcased in a Venn diagram

Based on SRGs, WGCNA analysis was performed with an optimal soft-threshold power set to 7 to ensure a scale-free network structure (R^2^ = 0.9) (Fig. 1A). Seven modules were created by clustering genes with analogous expression patterns through a dendrogram analysis (Fig. 1B,C). Within these various modules, the turquoise module, which comprised 2150 genes, displayed the most robust correlation, exhibiting an ME of 0.51 and a significance level of *p* =8 × 10^−23^, and was therefore selected as the hub module. Differential expression analysis based on TCGA-LIHC identified 1595 DEGs, including 1530 upregulated and 65 downregulated genes (Fig. 1D). Finally, 1004 DEGs with potential relevance to HCC were identified through cross-validation (Fig. 1E).

### 3.2 Validation of the Prognostic Model

**Figure 2:**
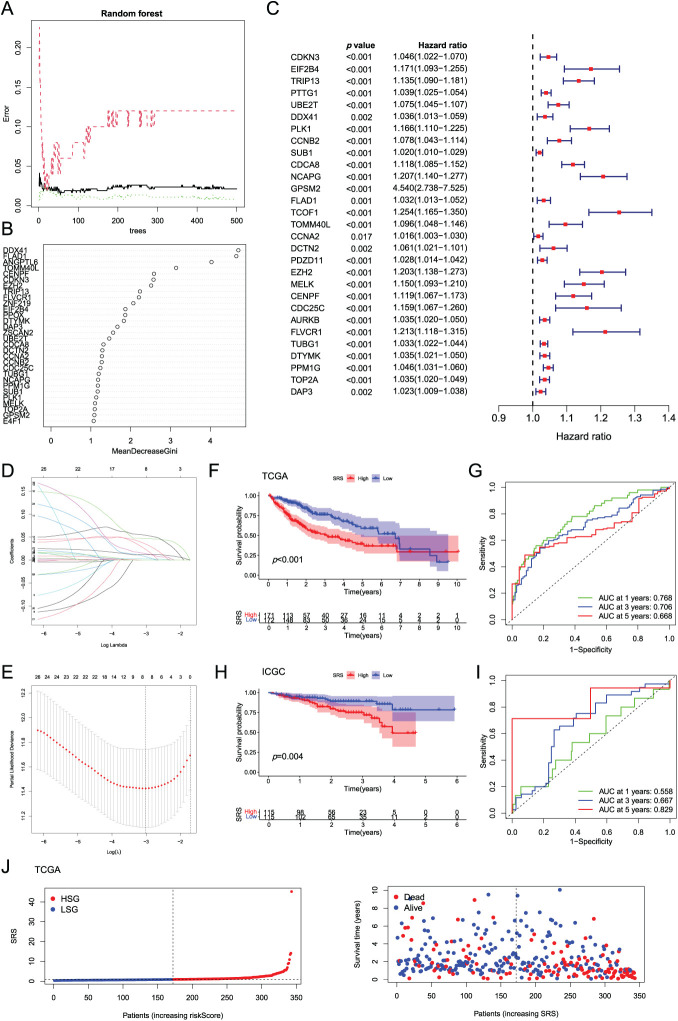

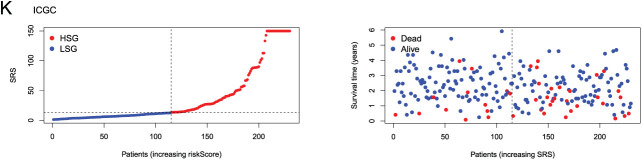
Construction and validation of the SRS. (**A**) Random forest plot filtering key genes; (**B**) MeanDecreaseGini showing the contribution of features in the random forest model; (**C**) Examination of key genes through unv-Cox regression analysis; (**D, E**) LASSO analysis; (**F–I**) Utilization of Kaplan-Meier survival curves and ROC curves to assess the SRS in the TCGA dataset and the ICGC databas; (**J, K**) Displaying the distribution of SRS and survival status and time in both the TCGA and ICGC cohorts

Random forest analysis filtered and selected 34 genes with an importance score greater than 1 (Fig. 2A). Following additional screening reliant on gene contribution, 29 genes were discerned to be notably linked to HCC prognosis, as demonstrated in Fig. 2B. Subsequently, unv-Cox, LASSO, and mut-Cox analyses were performed to choose and construct models (Fig. 2C–E), resulting in a prognostic SRS model comprising four SRGs: SRS = (EIF2B4 expression ∗ 0.093) + (CDCA8 expression ∗ 0.069) + (TCOF1 expression ∗ 0.108) + (T0MM40L expression ∗ 0.077).

The model’s predictive efficacy was investigated through analysis of data from the TCGA-LIHC and ICGC-LIHC databases. Survival outcomes worsened with high SRS group (HSG) as validated by Kaplan-Meier survival curves, showcasing the association between them (Fig. 2F,H). Analysis of ROC curves from the TCGA database demonstrated the predictive precision of the model, showcasing AUC values of 0.768, 0.706, and 0.668 for survival at 1-, 3-, 5-year, correspondingly (Fig. 2G). ROC curve analysis based on the ICGC database showed AUCs of 0.558, 0.667, and 0.829 for 1-, 3-, 5-year survival, respectively (Fig. 2I). Additionally, visual representations demonstrated that HSG correlated with shorter survival times (Fig. 2J,K).

### 3.3 Examination of Clinical Factors in HCC Patients

**Figure 3:**
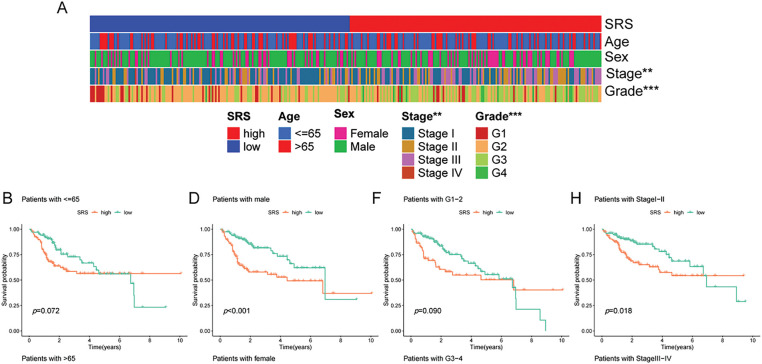

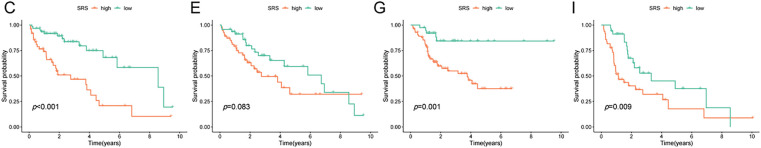
Exploration of the relationship between SRS and clinicopathological attributes. (**A**) Visualization of clinical traits distribution in HSG and LSG; (**B, C**) Evaluation of survival patterns among varied age groups through Kaplan-Meier analysis; (**D, E**) Evaluation of survival patterns among varied sex groups through Kaplan-Meier analysis; (**F, G**) Evaluation of survival patterns among varied grade groups through Kaplan-Meier analysis; (**H, I**) Evaluation of survival patterns among varied tumor stage groups through Kaplan-Meier analysis. ** indicates *p* < 0.01; and *** indicates *p* < 0.001

An extensive review of clinical parameters in HCC revealed significant differences in tumor stage and pathological grade between different SRS categories (Fig. 3A). Moreover, the prognostic efficacy of the SRS was affirmed within diverse pathological profiles of the patients. The data illustrated that individual in the HSG group experienced poorer overall survival (OS) compared to those in the low SRS group (LSG) across a range of clinical parameters (age, sex, pathological grade, and tumor stage, Fig. 3B–I.

### 3.4 Enrichment Analysis

**Figure 4:**
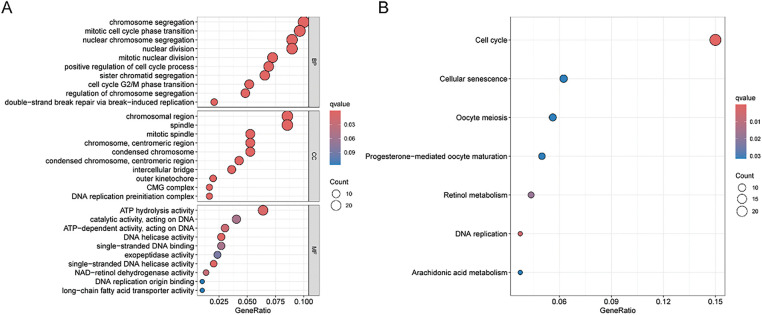

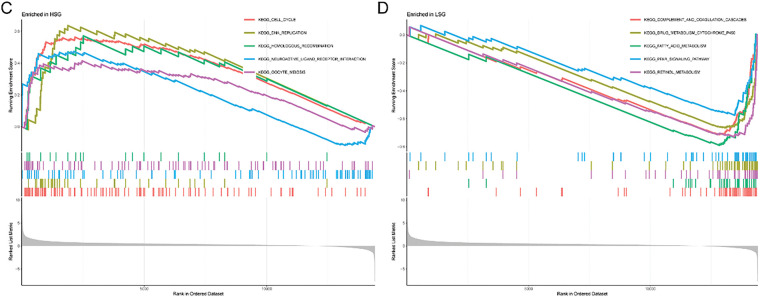
GO, KEGG, and GSEA analyses. (**A**) Illustration of GO analysis results through a bubble plot; (**B**) Illustration of KEGG analysis results through a bubble plot; (**C, D**) Application of GSEA analysis within HRG and LRG

First, the examination involved analyzing gene expression variances between the high-risk and low-risk sets (|log_2_FC| > 1, FDR < 0.05). Subsequent investigations comprised conducting GO and KEGG analyses utilizing the identified SRGs to delve into their biological attributes. The GO analysis highlighted the predominant enrichment of DEGs in chromosome segregation (Fig. 4A). Furthermore, KEGG analysis (Fig. 4B) revealed significant enrichment of DEGs in cell cycle. Notably, they also exhibited enrichment in the arachidonic acid metabolism pathway. GSEA results for the HSG indicated significant enrichment in cell cycle (Fig. 4C). For the LSG, GSEA results indicated significant enrichment in complement and coagulation cascades (Fig. 4D).

### 3.5 Construction a Nomogram Based on the SRS

**Figure 5:**
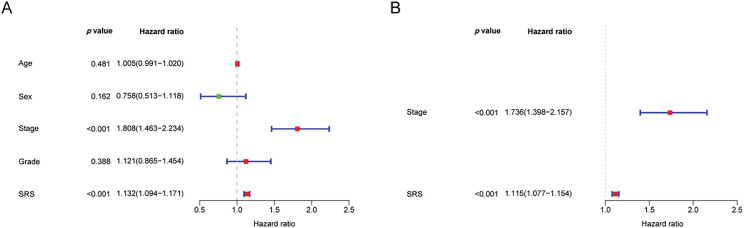

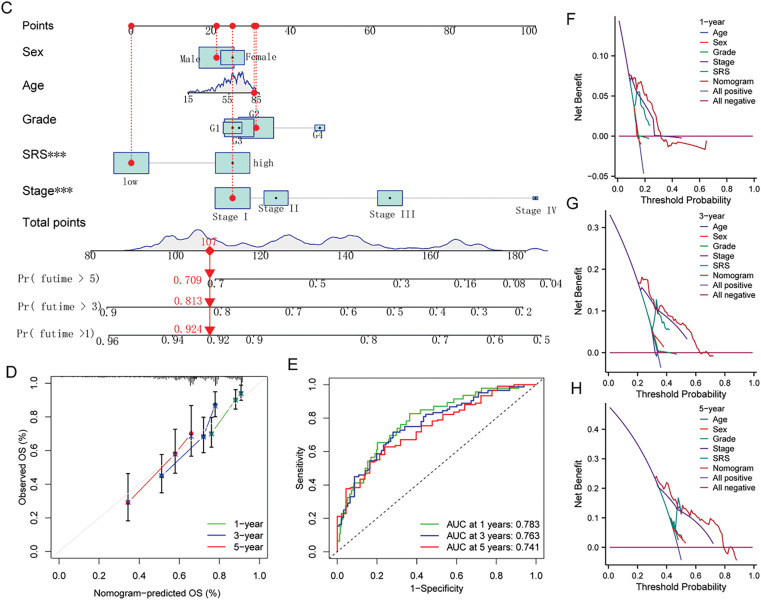
Construction of the nomogram. (**A, B**) Forest plots depicting the results of unv-, mut-Cox regression analyses; (**C**) Construction of the nomogram combining common clinical parameters and SRS; (**D, E**) Nomogram’s calibration curve and ROC curve; (**F–H**) Nomogram’s Decision curve. ***, *p* < 0.001

To develop a more practical and stable clinical prediction tool, a nomogram was constructed by integrating multiple common clinicopathological parameter. Analysis via unv-Cox regression indicated that tumor stage, pathological grade, and the SRS emerged as prognostic risk factors for HCC (Fig. 5A). Following Multivariate Cox regression analysis, it was affirmed that tumor stage and the SRS retained their autonomy as prognostic factors for HCC, even after accounting for various clinicopathological variables (Fig. 5B). A nomogram was then constructed by combining common clinicopathological parameters with SRS (Fig. 5C). Calibration curves and ROC curves demonstrated excellent accuracy and robustness in predicting 1-, 3-, 5-year survival rates (Fig. 5D,E). According to the decision curve analysis, the nomogram exhibited enhanced clinical advantages when forecasting the 1-, 3-, and 5-year prognoses (Fig. 5F–H).

### 3.6 The Correlation of SRS with the Immune Microenvironment

**Figure 6:**
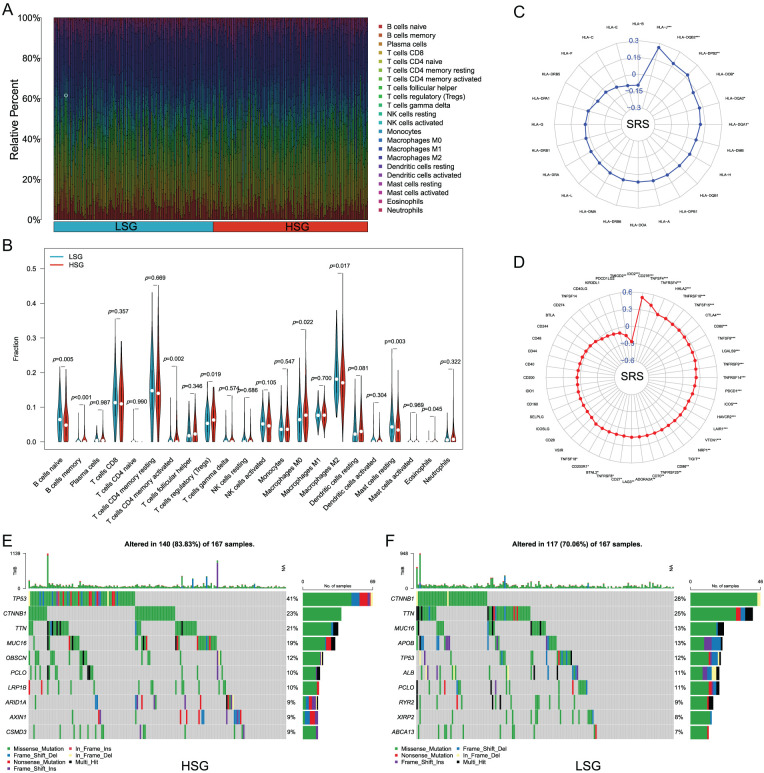
Analyzing the correlation between SRS and the tumor microenvironment involves. (**A, B**) Examining various types of immune cell enrichment in groups with HSG and LSG; (**C**) Investigating the relationship between SRS and ICM; (**D**) Exploring the association between SRS and molecules HLA molecules; (**E**) Identifying the top ten mutated genes within the HSG; (**F**) Identifying the most prevalent mutated genes within the LSG. *, *p* < 0.05; **, *p* < 0.01; and ***, *p* < 0.001

Upon executing CIBERSORT, the heatmap showed that with increasing SRS, the proportions of M2 macrophages and naive B cells decreased, while the proportions of regulatory T cells and M0 macrophages increased (Fig. 6A). The Wilcoxon test additionally validated the greater abundance of M0 macrophages and regulatory T cells within the HSG. In contrast, M2 macrophages and naive B cells, exhibited higher abundance in the LSG, suggesting a plausible association between the SRS and the immune environment of HCC (Fig. 6B).

Acknowledging the significance of HLA molecules and immune checkpoint molecules (ICM) in immunotherapeutic contexts, we examined the relationship between the SRS and 24 HLA molecules along with 48 ICM. Findings unveiled a notable positive correlation between the SRS and 31 ICM, as well as 6 HLA molecules (Fig. 6C,D). Additionally, the examination of somatic mutation profiles among HCC patients unveiled a substantial elevation in the TP53 mutation frequency within the HRG compared to the LSG (Fig. 6E,F).

### 3.7 Chemotherapy Sensitivity in HCC

**Figure 7:**
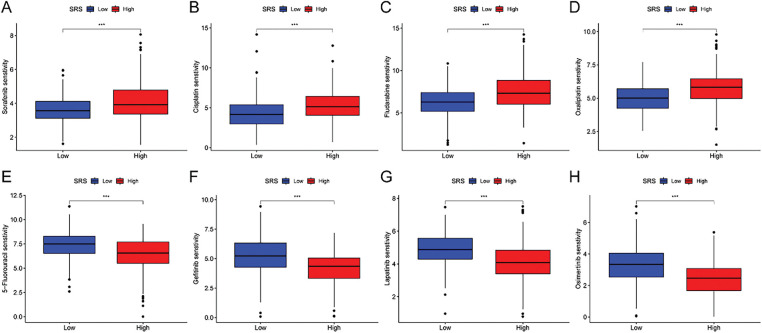
Correlation analysis between SRS and sensitivity to common drugs. (**A–H**) Differences in IC_50_ values of eight common drugs between HSG and LSG. ***, *p* < 0.001

Furthermore, we estimated the IC_50_ values of several drugs using predictive algorithms and compared the chemotherapy sensitivity between HRG and LSG. The disparities in IC_50_ values for sorafenib, cisplatin, fludarabine, oxaliplatin, 5-fluorouracil, gefitinib, lapatinib, and osimertinib among the two groups. Specifically, the IC_50_ values of sorafenib, cisplatin, fludarabine, and oxaliplatin were lower in the LSG (Fig. 7A–D), indicating that these drugs are more effective for LSG. Conversely, the IC_50_ values of 5-fluorouracil, gefitinib, lapatinib, and osimertinib were lower in the HSG (Fig. 7E–H), suggesting that these drugs are more suitable for HSG.

### 3.8 Identification of TOMM40L as a Key Gene

**Figure 8:**
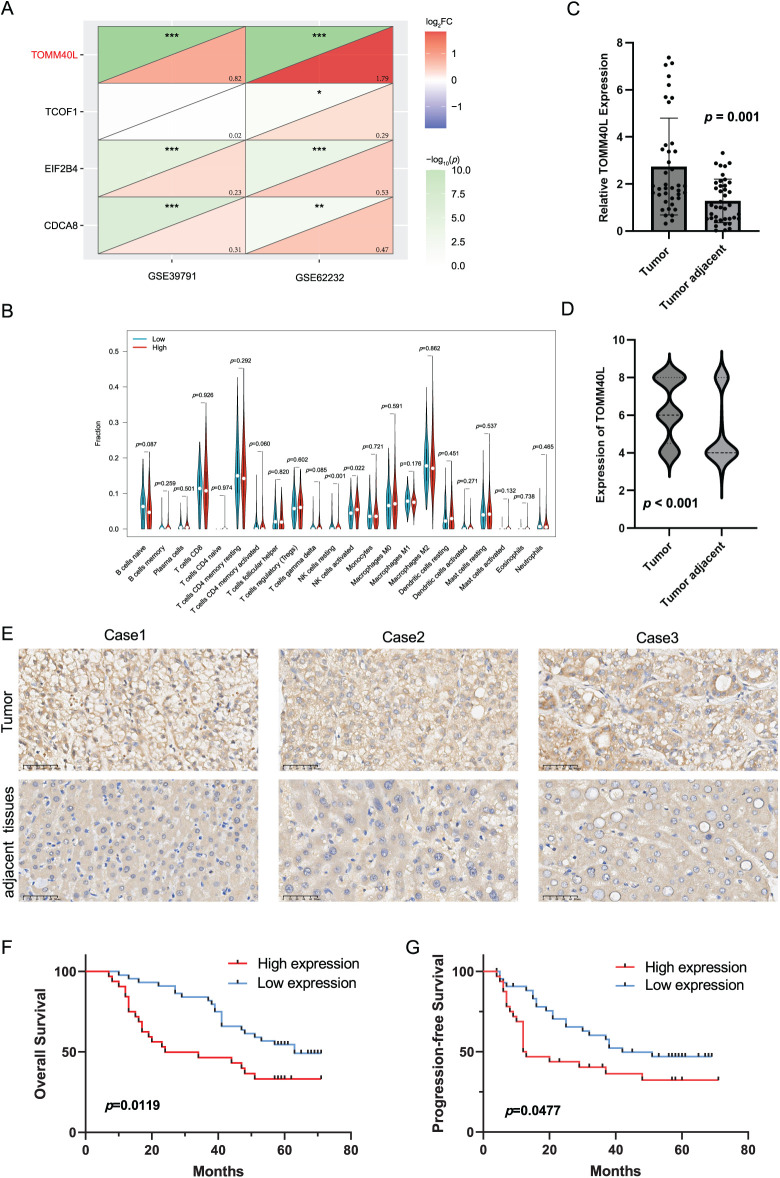
The expression patterns and prognostic significance of TOMM40L in HCC. (**A**) Differential analysis screening core gene TOMM40L; (**B**) various immune cells in high and low expression of TOMM40L; (**C, D**) mRNA and protein levels of TOMM40L in HCC tissues (*n* = 76); (**E**) Representative IHC images of TOMM40L protein expression (400×); (**F, G**) The assessment of TOMM40L’s prognostic significance through Kaplan-Meier analysis. *, *p* < 0.05; **, *p* < 0.01; and ***, *p* < 0.001

Based on two datasets (GSE39791 and GSE62232) from the GEO database, the model genes in HCC were compared, revealing that the expression of gene TOMM40L was significantly increased in HCC and had a larger change magnitude than the other three genes (Fig. 8A). Therefore, it was selected as the core gene for further study. Upon further examination of its association with immune infiltrating cells, it was found the high expression group of the TOMM40L demonstrated notably elevated levels of both resting and activated NK cells compared to the low expression group (Fig. 8B).

### 3.9 Assessing the Expression Levels of TOMM40L in HCC and Its Correlation with Clinical Outcomes

We further discussed the expression of TOMM40L in HCC tissues. We found that the levels of TOMM40L in tumor tissues were significantly increased at both the mRNA and protein levels (Fig. 8C–E). After categorizing 76 HCC cases into groups based on TOMM40L protein levels in tumor tissues, a low expression group and a high expression group were established. Kaplan–Meier survival analysis revealed notably reduced 5-year OS and progression-free survival (PFS) in the high expression group in contrast to the low expression group (Fig. 8F,G). Analysis of the relationship between TOMM40L protein levels in HCC tissues and 13 key clinicopathological characteristics revealed significant correlations with patient survival time, clinical stage, T stage, and Gamma-Glutamyl Transferase (GGT) levels (Table 2).

**Table 2:** Correlation analysis between TOMM40L protein levels in HCC and clinicopathological parameters in 76 HCC cases

Variable	Spearman correlation coefficient (r)	*p*
Survival time	−2.98	0.009
Age	−0.022	0.851
T stage	0.311	0.006
Clinical stage	0.283	0.013
pathology grade	0.022	0.849
Tumor number	−0.012	0.915
Tumor size	0.192	0.097
Cirrhosis nodules	0.118	0.310
ALT	0.098	0.399
ALB	−0.053	0.649
AFP	−0.226	0.050
TBil	−0.151	0.194
GGT	0.369	0.001

Note: ALT: Alanine Aminotransferase; ALB: Albumin; AFP: Alpha-Fetoprotein; TBil: Total Bilirubin; GGT: Gamma-Glutamyl Transferase.

The comparison of TOMM40L protein levels based on clinicopathological parameters revealed that the levels were significantly higher in males than females. Additionally, TOMM40L protein levels were elevated in patients at clinical stage II compared to those at stage I, and in T2N0M0 compared to T1N0M0 (Table 3).

**Table 3:** The correlation between TOMM40L protein levels in cancer tissues of 76 HCC patients and clinical pathological features

Characteristics	Case	TOMM40L expression M (min, max)	*p*
All cases	76		
Intra-tumor	76	6 (4, 8)	<0.001
Peri-tumor	76	4 (3, 8)	
Age (years)			
<60	57	6 (4, 8)	0.844
≥60	19	6 (4, 8)	
Sex			
Male	64	6 (4, 8)	0.005
Female	12	4 (4, 8)	
Pathological grading			
II	53	6 (4, 8)	0.084
II&IIII	23	6 (4, 8)	
Liver cirrhosis			
Yes	68	6 (4, 8)	0.559
No	8	7 (4, 8)	
Hepatic cirrhotic nodules (mm)			
≤3	31	6 (4, 8)	0.311
>3	33	6 (4, 8)	
No	12	6 (4, 8)	
Tumor capsule			
Yes	41	6 (4, 8)	0.860
No	35	6 (4, 8)	
Clinical staging			
1	56	6 (4, 8)	0.014
2	20	8 (4, 8)	
TNM staging			
T1N0M0	55	6 (4, 8)	0.007
T2N0M0	21	8 (4, 8)	
Recurrence			
Yes	47	6 (4, 8)	0.391
No	29	6 (4, 8)	
HBsAg			
+	65	6 (4, 8)	0.481
–	11	6 (4, 8)	
Tumour size (cm)			
<5	37	6 (4, 8)	0.834
≥5	39	6 (4, 8)	
ALT (U/L)			
≤40	38	6 (4, 8)	0.054
>40	38	7 (4, 8)	
ALB (g/L)			
≥40	16	6 (4, 8)	0.670
<40	60	6 (4, 8)	
AFP (ng/mL)			
<20	26	6 (4, 8)	0.255
20~200	17	8 (4, 8)	
201~1000	14	6 (4, 8)	
≥1000	19	6 (4, 8)	
TBil (μmol/L)			
≤17.1	57	6 (4, 8)	0.070
>17.1	19	6 (4, 8)	

Note: TNM: Tumor, Node, Metastasis; ALT: Alanine Aminotransferase; ALB: Albumin; AFP: Alpha-Fetoprotein; TBil: Total Bilirubin.

### 3.10 The Impact of TOMM40L Gene Expression on the Malignant Phenotype of HCC Cells

**Figure 9:**
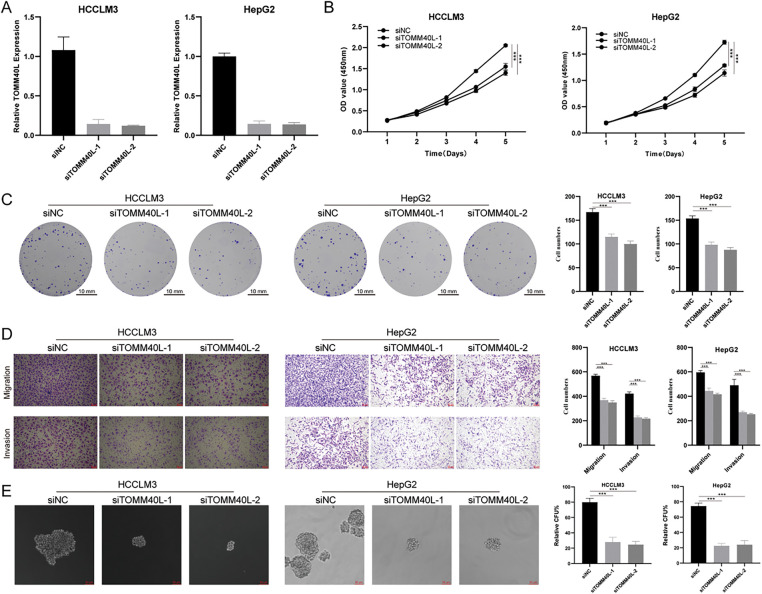
*In vitro* impact of TOMM40L regulation on the carcinogenic properties of HCC cells. (**A**) Knockdown efficiency of TOMM40L in HCCLM3 and HepG2 cells; (**B, C**) CCK8 (*n* = 5) and colony formation assay (*n* = 3) showing the effect of siTOMM40L on proliferation capacity of HCCLM3 and HepG2 cells; (**D**) Transwell assay showing the effect of siTOMM40L on migration and invasion of HCC cells (*n* = 3); (**E**) Spheroid assay analysis showed the effect of siTOMM40L on the spheroid formation ability of HCC cells (*n* = 3). ***, *p* < 0.001

To explore the effects of TOMM40L on the functions of HCC cells *in vitro*, we constructed HCC cell lines HCCLM3 and HepG2 with silenced TOMM40L expression. Both siTOMM40L-1 and siTOMM40L-2 significantly inhibited the expression of TOMM40L in HCCLM3 and HepG2 cells (Fig. 9A). Findings from the CCK8 and colony formation tests demonstrated that diminishing TOMM40L levels led to a decrease in proliferative capacity (Fig. 9B,C). Furthermore, the Transwell assays demonstrated that the reduction of TOMM40L also reduced the migratory and invasive capacity (Fig. 9D). Finally, the sphere formation experiment showed that knockdown of TOMM40L effectively inhibited the sphere-forming ability of HCC cell lines HCCLM3 and HEPG2 (Fig. 9E).

## 4 Discussion

The prognosis for advanced HCC is frequently unfavorable, primarily attributed to widespread metastasis and severe complications [16]. Therefore, early detection of HCC is crucial for improving clinical outcomes. Current HCC screening methods (e.g., ultrasound, AFP, abnormal prothrombin, predictive models, and liquid biopsy) generally lack sufficient sensitivity and specificity [17]. Although liver biopsy plays an important role in diagnosis, its invasiveness limits widespread application. Thus, identifying reliable early detection biomarkers for HCC is essential to improve diagnostic accuracy and prognosis.

The development of novel biomarkers and predictive models promises to advance early diagnosis and prognosis assessment of HCC, enabling timely intervention to improve outcomes. Systematic analysis of specific gene sets has made significant progress in predicting cancer prognosis. Stemness gene models have been reported to predict HCC prognosis [18–20], but these models have not significantly improved survival rates. Hence, we developed a more precise stemness-related gene model for HCC prognosis. After validation in TCGA and ICGC cohorts, the SRS highlighted that the HSG exhibited notably poorer outcomes in contrast to the LSG. Mut-Cox analysis identified the SRS stood as an independent prognostic determinant for HCC. ROC curve analysis additionally illustrated that the SRS surpassed alternative clinicopathological markers in prognostic forecasting. A more pragmatic nomogram was formulated and evaluated utilizing the SRS.

We performed an in-depth analysis of DEGs between HSG and LSG. Using GO, KEGG, and GSEA unveiled variances in enriched pathways across the distinct groups. HLA molecules and ICK are critical predictors of immunotherapy response [21–25]. The analysis indicated a positive correlation between the SRS and the majority of HLA molecules and ICM, suggesting its potential in predicting immunotherapy response. Notable variances in immune-associated cells between the HSG and LSG suggest diverse reactions to immunotherapy. Specifically, the HSG exhibited a significantly elevated TP53 mutation frequency, potentially influencing the unfavorable prognosis [26]. Furthermore, the sensitivity of SRS subcategories to eight prevalent chemotherapeutic medications was evaluated, providing a basis for clinical decision-making.

Regarding the relationship between the four SRGs in the SRS model and HCC, studies have shown that cell division cycle associated 8 (CDCA8) may act in HCC by activating the cell cycle and E2F-related pathways [27]. In addition, CDCA8 can affect HCC stemness by regulating the AKT/β-Catenin signaling pathway [28]. Treacher collins-franceschetti syndrome 1 (TCOF1) promotes HCC tumorigenesis by coordinating oncogene activation and rRNA generation [29]. It is worth mentioning that TCOF1, as an SRG, was used in another study to construct a model to predict HCC prognosis [30]. Eukaryotic translation initiation factor 2B, subunit 5 epsilon, (EIF2B5) is highly expressed in lung and breast cancers [31] and is also a biomarker for colorectal cancer [32]. The relationship between EIF2B4, a subtype of EIF2B, and HCC mechanisms is still unclear. In ovarian cancer, high TOM40 expression is associated with poor prognosis, promoting cancer growth by regulating mitochondrial activity and ATP levels [33]. TOMM40L gene is a mitochondrial outer membrane subunit involved in the composition of the mitochondrial outer membrane translocation complex [34]. The expression level of TOMM40L gene was upregulated in gemcitabine-resistant pancreatic cancer cell lines in a time-dependent manner, thus showing its potential as a candidate biomarker for diagnosis and treatment [35]. Although no relevant reports have been found in HCC, it may be involved in disease progression by affecting mitochondrial activity.

Analysis of immune infiltration indicated the high TOMM40L expression displayed notably elevated levels of resting and activated NK cells in contrast to the low expression group. NK cells are crucial in the immune system, capable of recognizing and killing infected or tumor cells [36,37]. Elevated resting NK cell levels in the high expression group may weaken immune surveillance, promoting HCC cell proliferation, whereas high activated NK cell levels might be a response to inhibit tumor proliferation.

Moreover, elevated TOMM40L mRNA and protein levels were observed in HCC tissues, showing a significant negative correlation with the OS and PFS of patients with HCC. Additionally, TOMM40L protein levels were closely associated with clinical and pathological stages of HCC. Male patients had higher TOMM40L protein levels than female patients, suggesting worse prognosis for males, consistent with studies indicating higher recurrence risk post-surgery in male liver cancer patients [38]. This may involve factors such as sex hormone levels [39], genetic factors, and lifestyle differences. GGT is an enzyme expressed in the liver, often elevated in HCC and other liver diseases. High GGT levels indicate disease progression and poor prognosis, participating in HCC progression through pathways like inducing DNA damage, releasing reactive oxygen species, blocking chemotherapy, and regulating microRNAs [40–43]. The significant positive correlation between TOMM40L protein and GGT levels suggests an association with poor HCC prognosis. In addition, we conducted an in-depth exploration of the biological role of the TOMM40L gene. We found that TOMM40L has the ability to promote HCC cell progression and stemness, indicating that it plays an oncogenic role in HCC and has the potential to be a therapeutic target. In the future, nano-RNA therapy targeting TOMM40L will provide a theoretical basis for clinical decision-making in the treatment of HCC with TOMM40L targeting [44].

There are still some limitations in this study. First, the sample size lacks the sample size from a real-world cohort, which may limit the generalizability of our findings to different HCC populations. Future studies with larger, multi-center cohorts representing a broader range of patient demographics are needed to validate the robustness and generalizability of the prognostic model. Secondly, the study employed qPCR and IHC techniques, which have inherent limitations. Discrepancies between mRNA and protein expression levels are also possible. To further validate our findings, future studies should utilize more robust quantitative methods for assessing gene and protein expression. These limitations notwithstanding, our findings provide valuable insights into HCC prognosis and warrant further investigation.

## 5 Conclusion

The development of the SRS model introduces a novel avenue for evaluating HCC prognostication and offers crucial insights for personalized clinical interventions. Furthermore, significant upregulation of key SRGs like TOMM40L in HCC, in contrast to adjacent non-tumor tissues, was observed. This elevation correlated strongly with adverse OS, PFS, and diverse clinical parameters (including TNM and clinical staging, GGT levels, and sex). Of utmost significance, the oncogenic function of TOMM40L in HCC was confirmed through meticulous *in vitro* cell assays.

## Data Availability

The original contributions presented in the study are included in the article. Further inquiries can be directed to the corresponding author.

## Abbreviations

HCC: Hepatocellular carcinoma
SRGs: Stemness-related genes
SRS: Stemness-related scores
CSCs: Cancer stem cells
DEGs: Differentially expressed genes
unv-Cox: Univariate Cox
mut-Cox: Multivariate Cox
HSG: High SRS group
LSG: Ligh SRS group
ICM: Immune checkpoint molecules
OS: Overall survival
PFS: Progression-free survival
EGF: Epidermal growth factor
FGF: Fibroblast growth factor
TNM: Tumor, Node, Metastasis
ALT: Alanine Aminotransferase
ALB: Albumin
AFP: Alpha-Fetoprotein
TBil: Total Bilirubin
GGT: Gamma-Glutamyl Transferase
CDCA8: Cell Division Cycle Associated 8
TCOF1: Treacher Collins-Franceschetti Syndrome 1
EIF2B5: Eukaryotic Translation Initiation Factor 2B, subunit 5 epsilon
